# Functional reconstruction of complex arm defect with modified Capanna technique and latissimus dorsi muscle transfer: case report and literature review

**DOI:** 10.1016/j.xrrt.2025.100656

**Published:** 2025-12-29

**Authors:** Pedro Machado, Francisco Carvalho, Maria Clara Correia, Gonçalo Gandra, Filipe Duarte, Inês Ínsua, Ruben Coelho, Joana Costa, Ricardo Horta

**Affiliations:** a Unidade de Saúde Local São João, Porto, Portugal; b Faculty of Medicine – University of Porto Porto, Portugal

**Keywords:** Modified capanna technique, Humerus reconstruction, Free fibula flap, Latissimus dorsi muscle transfer, Complex arm reconstruction, Orthoplastic

Limb trauma can have devastating consequences, especially when it results in the destruction of multiple tissues, which can, in extreme cases, compromise the vascularization and viability of the limb. Following initial management, including patient resuscitation and stabilization and, if needed, surgical stabilization of the damaged limb, the surgical team faces challenges such as decisions regarding limb salvage vs. amputation, surgical timing, and reconstructive methods. The primary goal is to restore, as much as possible, the function of the limb.

We present a clinical case of a polytrauma patient with severe mangling of the proximal upper limb, along with our line of reasoning, the literature on which we based our approach, and the reconstructive process, tissue by tissue.

## Methods—case report

**Figure 1:**
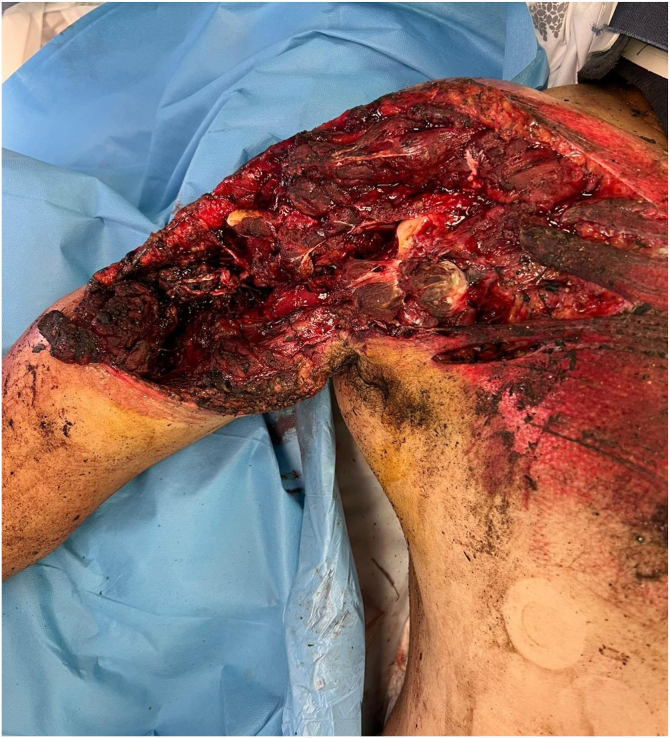
Upon admission to the emergency room.

A 51-year-old man with no medical history was admitted after a workplace backhoe accident. *He* sustained a severe crush injury to the right upper limb, classified as Gustilo–Anderson IIIB, with extensive soft tissue destruction at the shoulder and anterior arm (Fig. 1). It is important to note that the patient has a history of severe burn on the posterior aspect of this arm, resulting in scar tissue and consequently reduced skin elasticity.

The Mangled Extremity Severity Score (MESS) was 6, scoring points for maintained limb perfusion (1 point), age over 50 (2 points), and high-energy mechanism (3 points).

The patient was intubated at the accident site, preventing preoperative neurological assessment, but distal pulses confirmed perfusion. Additional injuries included rib fractures with pulmonary contusion, a unicortical mandibular fracture (both treated conservatively), ocular trauma requiring multiple surgeries, and left forearm degloving injury with skin and extensor tendon loss.

**Figure 2:**
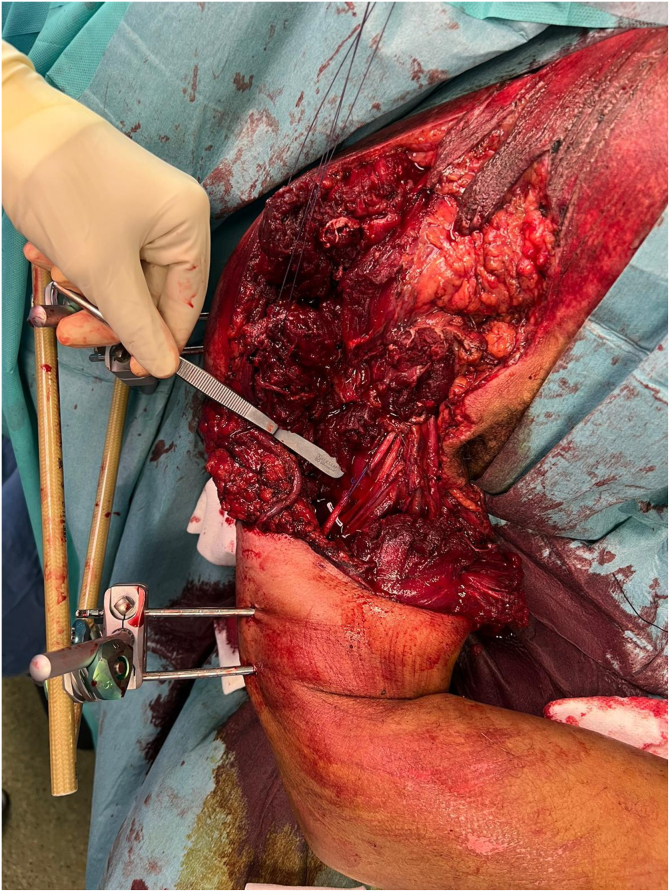
After external fixation and thorough débridement.

Emergency surgery involved wound débridement and humeral external fixation, revealing an 11-cm bone defect (Fig. 2). A 5-cm radial nerve gap, anterior arm muscle damage, and extensive skin loss were also noted. The median and ulnar nerves, as well as the brachial artery, although exposed, remained intact. The left forearm underwent tendon repair and flap approximation.

**Figure 3:**
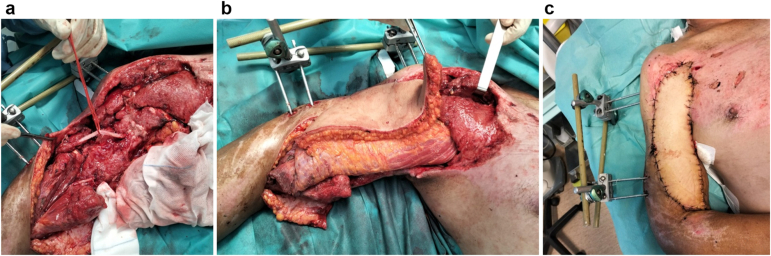
(**a**) Reconstruction of radial nerve defect with 5 sural nerve cables. (**b**) and (**c**) Bipolar latissimus dorsi muscle transfer with myocutaneus flap.

Postoperative care included staged wound management. A second-look surgery at 48 hours involved further débridement and negative pressure wound therapy. Ten days later, the radial nerve was reconstructed with five sural nerve graft cables, and a pedicled latissimus dorsi myocutaneous flap with bipolar muscle transfer restored elbow flexion (Fig. 3). The muscle origin was fixed to the coracoid process using two DePuy Mitek 2.0 (DePuy Synthes, Raynham, MA, USA). Distally, the muscle was sutured to the remaining tendinous insertion of the bicipital muscle at its radial tuberosity. No complications occurred.

**Figure 4:**
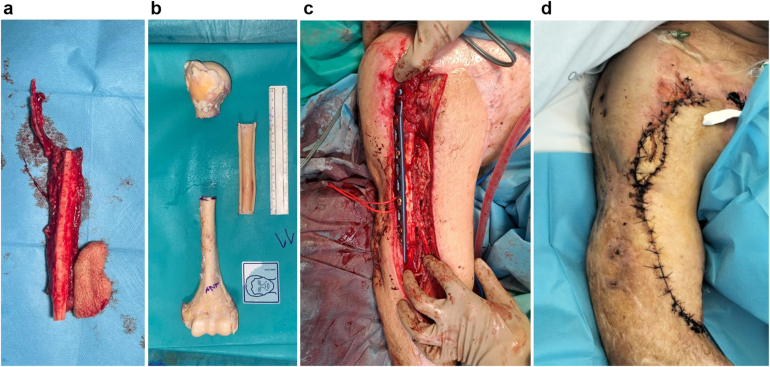
Bone reconstruction with a free osteoseptocutaneous fibula flap (**a**), using a cadaveric humerus graft (b) through the combined Capanna technique (**c**). Final result in (**d**).

**Figure 5:**
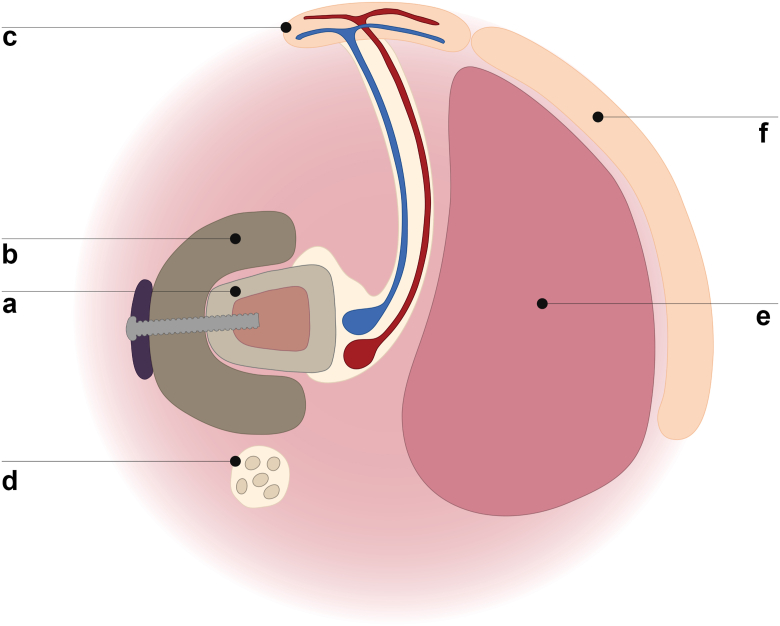
Schematic representation of tissue reconstruction: (a) fibula bone flap with cadaveric graft support, (b) *Capanna* technique and fibula flap–corresponding skin island, (c) radial nerve reconstruction using five sural nerve graft cables (d), and latissimus dorsi flap (e) with its skin island (f).

The patient was hospitalized for three months due to ophthalmologic procedures and prolonged intravenous antibiotics. Four months postaccident, elective surgery reconstructed the 11-cm humeral defect using a free fibula osteoseptocutaneous flap combined with a cadaveric allograft (Capanna technique) (Figs. 4 and 5). Vascular anastomoses were performed T-T to the radial artery and cephalic vein. The anastomosis to the radial artery required its dissection down to the wrist and its retroflexion toward the arm. A minor wound dehiscence resolved with flap advancement. The patient was discharged with good recovery and referred for physiotherapy.

## Results

**Figure 6:**
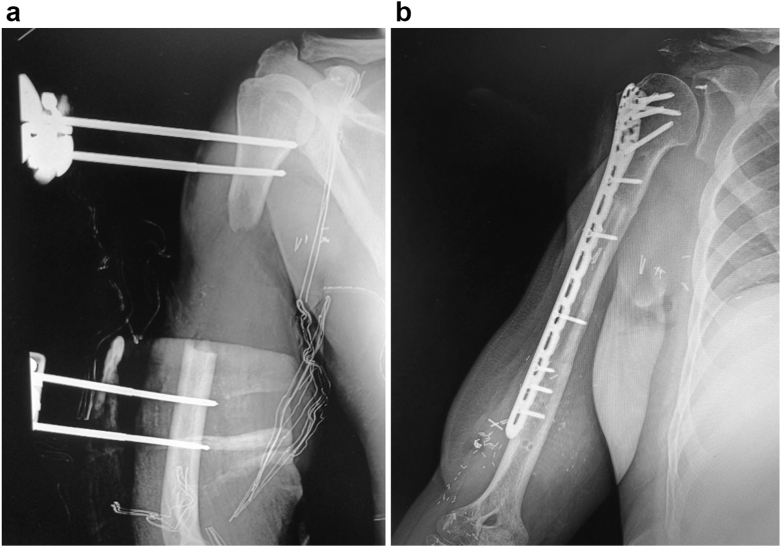
Segmental defect of the diaphysis preoperatively (**a**) and good consolidation at 9 months post–bone reconstruction (**b**).

**Figure 7:**
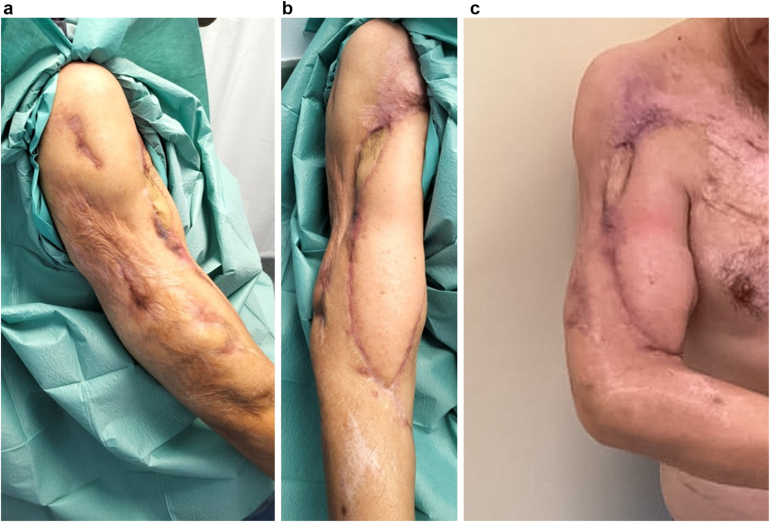
Outcome 9 months postoperatively. (**a**) Lateroposterior view highlighting scar tissue from a previous burn; (**b**) anterior view showing the cutaneous island of the peroneal flap and the latissimus dorsi flap; and (**c**) active elbow flexion at 90°.

Nine months after bone reconstruction and twelve months after nerve and muscle reconstruction, the patient presents with good healing. Radiographs show complete consolidation 9 months after surgery (Fig. 6). There is good mobility of the elbow, with active flexion up to 90° [M5/5], pronation, and supination (Fig. 7). Regarding the hand and wrist, there is recovery of wrist extension (M3/5), with no changes in flexion. The fingers extend, but mainly due to the function of the interossei. Finger flexion remains unchanged.

Sensitivity in the territory of the radial nerve has recovered, limited to rough and protective sensation, and wrist extension is noted [M3/5]. The patient is satisfied with the final result, having preserved his independence and reasonable mobility of his dominant limb.

## Discussion

### Initial evaluation of the trauma patient

The initial step in managing a polytrauma patient should always be stabilization and resuscitation, followed by a thorough evaluation of the mutilated segments. The assessment should focus on determining the extent of damage to soft tissues, bone, neural structures, and blood vessels, and the resulting tissue vascularization. Damage to at least 3 of these 4 components compromises the viability of the limb, referred to as mangled limb, which imposes urgent surgical intervention.^25^^,^^32^

### Limb salvage vs. amputation

In emergency cases, the trauma team often faces the dilemma of saving a mutilated limb or opting for amputation. This decision can sometimes be straightforward due to the high level of tissue destruction and the location of the injury. In destructive and proximal limb injuries, the evaluation should be multidisciplinary and take into account the patient's overall condition, autonomy, and potential for recovery and rehabilitation.^4^^,^^5^^,^^25^

Several scoring systems, including MESS, can help guide this decision. However, none provide definitive answers, and the final decision remains clinical.^11^^,^^22^ In this case, the MESS score of 6 fell on the threshold between salvage and amputation, thereby enhancing its limited utility in guiding decision-making in the emergency setting. There is no scientific consensus or perfect algorithm for managing a mutilated limb.

Despite extensive tissue destruction, the decision to proceed with limb reconstruction was based on hemodynamic stability, preservation of major vessels and nerves, maintained elbow extension, integrity of forearm and hand function, and the presence of additional complex injuries to the contralateral upper limb.

### Limb reconstruction

Due to advancements in surgical techniques, particularly microvascular solutions, today's surgeons have access to an array of approaches for virtually all types of defects. However, a case-by-case approach is recommended to ensure optimal outcomes.^4^^,^^19^^,^^25^

Assuming an attempt to salvage the limb, initial approach should include bone stabilization, typically using external fixation, along with extensive débridement and, if necessary, vascular repair. The threshold for reoperation for débridement and infection control should be low in these cases due to the high degree of local contamination.

The assessed bone defect of 11 cm exceeded the acceptable 5 cm of bone shortening in the humerus,^27^ mandating structural reconstruction of the limb. In the case described, the patient required two débridement surgeries and two five-day cycles of negative pressure therapy combined with irrigation using sodium hypochlorite solution prior to tissue rebuild.

Negative pressure therapy is a helpful adjunct for controlling infection, inflammation, and promoting healing in complex wounds, and its use is widely accepted and supported,^26^ provided it does not delay soft tissue coverage. It is particularly beneficial for polytrauma patients requiring further stabilization in the intensive care unit before major surgery.

### Timing for reconstruction

There is no consensus regarding the timing of reconstruction. Empirically, it is generally believed, as advocated by various authors,^6^^,^^17^^,^^18^^,^^23^ that there are two main strategies: either soft tissue coverage within the first 72 hours to prevent the onset of overt inflammatory and infectious phases, or waiting for stabilization between 2 and 3 weeks after the initial injury.

### Skin defect

Given a highly complex and contaminated wound with extensive soft tissue injury, achievement of clean wound bed and good soft tissue coverage is mandatory for proper healing and reconstruction of remaining structures such as bone, muscle, or nerve. Substantial defects from severe trauma may require transfer of distant tissues when local or regional destruction limits the use of local flaps, or when the defect is too large.^16^

In this case, there was need for extensive soft tissue coverage of the arm and shoulder, including bone and neurovascular coverage and occupation of dead space in the anterior arm compartment, as well as restoration of elbow flexion.

In light of this challenge, the simplest solution was chosen: bipolar transfer of the latissimus dorsi muscle using a pedicled myocutaneous flap. This approach enabled simultaneous soft tissue reconstruction and restoration of elbow flexor function.

### Nerve reconstruction

Nerve defects that cannot be primarily repaired, such as the one described on the radial nerve, require interposition nerve grafts. The sural nerve is commonly used as a donor due to its length, subcutaneous access, and relatively low morbidity.^3^^,^^14^

Notably, if the reconstruction had been performed in a single-stage procedure, the sural nerve could have been included as part of a composite flap with the free fibular flap. At 12 months postoperatively, the patient demonstrated partial recovery of radial nerve function, consistent with expectations for nerve regeneration.

### Muscle reconstruction – elbow flexion restoration

Elbow flexion can be restored using various techniques, ranging from nerve transfers to muscle transfers.^34^ Nerve transfers are reserved for nerve injuries and are widely used in brachial plexus lesions. In late plexus injuries or when muscle masses cannot be utilized due to oncologic resection, severe traumatic destruction, congenital absence, or muscle disease, muscle transfer is preferred.

When muscle transfer is required, the most commonly used techniques are the transfer of the latissimus dorsi, pectoralis major, triceps brachii, and *Steindler* flexorplasty, with the first options generally being prioritized.^21^ When no local options exist, free muscle transfer is necessary, with *latissimus dorsi* and *gracillis* being the most commonly used, followed by the rectus femoris and medial *gastrocnemius*.^29^^,^^33^

In this case, the pedicled *latissimus dorsi* muscle was chosen due to its long vascular pedicle, ease of harvest, minimal donor-site morbidity, and robust and sizable skin paddle for soft tissue reconstruction. The bipolar transfer—where the muscle is proximally fixed at the coracoid process and distally at the radial tuberosity—stabilizes the shoulder while allowing for improved flexion of both the arm and elbow through a direct line of pull.^9^

### Bone reconstruction

After adequate soft tissue coverage, bone reconstruction was performed. Various techniques exist for bone defect repair, including distraction osteogenesis, the *Masquelet* technique with bone grafts, cadaver grafts, prosthetics, and bone flaps. A detailed discussion is beyond the scope of this paper, but it is consensual in literature that, when feasible, bone flaps are an appropriate choice for bone defects larger than 6 cm.^1^^,^^24^

The choice of bone flap reconstruction was based on the patient's condition, defect etiology, contamination risk, and defect size, as it provides vascularized tissue to a nonideal bed. Nonvascularized options can be risky in compromised tissue beds.^15^ Fibula bone flaps are widely used for segmental reconstruction and are superior to grafts or prostheses.^5^^,^^12^^,^^36^

Liaw et al^20^ reviewed 25 studies on upper limb traumatic reconstruction using free fibular flaps, concluding it to be safe and effective, and, due to its anatomical characteristics, it is the gold standard for autologous bone reconstruction of the upper limb. However, higher fracture risk at humerus occurs, likely due to biomechanical challenges of fixing the native humerus to the flap and the rotational forces involved in upper arm mobility.

The fibular bone flap may be used alone but can be insufficient, depending on the patient's age and physical condition. To increase strength and prevent fracture, it can be folded in a double-barrel configuration or combined with allograft, popularized by Capanna.^7^^,^^8^

These options are typically used for larger, load-bearing bones, such as those in the lower limb. However, considering the patient's age and potential for a return to physical activity, the combined technique was chosen.^37^ This provides mechanical stability and augmentation combined with biological properties of vascularized bone.^24^^,^^35^^,^^36^ A skin island aids monitoring of vascularization in the immediate postoperative period and adds healthy tissue to a bed typically traumatized with compromised soft tissues.

In this case, due to the extensive tissue destruction across the entire arm segment, the selection of recipient vessels for microsurgical transfer was not straightforward. Ultimately, radial artery was used up to the wrist, sacrificing a vascular axis.

### Similar cases

The literature presents cases primarily in an oncological context involving elective and planned resection of bone defects.^2^^,^^10^^,^^13^^,^^28^^,^^30^^,^^31^ This case is distinctive due to the extensive destruction of soft tissues and the need for the reconstruction of various tissue types. No reports were found regarding reconstructions of this scope in traumatic cases, where all tissue layers are involved, as seen in our report.

## Conclusion

This case underscores the complexity of managing severe polytrauma with extensive soft tissue and bone damage, highlighting the importance of an orthoplastic approach for successful limb salvage.

Despite the severity of the injury, the decision to preserve the limb was based on the patient's hemodynamic stability, maintained vascularization, and potential for functional recovery. The staged reconstructive process, utilizing advanced techniques such as negative pressure therapy, nerve grafting, muscle transfer, and free fibula flap, resulted in a favorable outcome with no complications.

The patient achieved reasonable mobility and functional recovery, emphasizing the essential postoperative rehabilitation to convert surgical success into therapeutic success, ensuring an optimized recovery for the patient.

## Disclaimers:

Funding: This paper received no specific grant from any funding agency, commercial, or not-for-profit sectors.

Conflicts of interest: The authors, their immediate families, and any research foundation with which they are affiliated have not received any financial payments or other benefits from any commercial entity related to the subject of this article.

Patient consent: Obtained.
